# Comparison of sutureless intrascleral fixation and sutured scleral fixation for the treatment of dislocated intraocular lenses

**DOI:** 10.1186/s12886-023-03020-1

**Published:** 2023-06-13

**Authors:** Yinglei Zhang, Yuan Zong, Xiangjia Zhu, Yi Lu, Chunhui Jiang

**Affiliations:** 1 Eye Institute, Department of Ophthalmology, Eye & ENT Hospital, Fudan University, Shanghai, 200031 China; 2 NHC Key Laboratory of Myopia (Fudan University), Key Laboratory of Myopia, Chinese Academy of Medical Sciences, Shanghai, 200031 China; 3 Shanghai Key Laboratory of Visual Impairment and Restoration, Shanghai, 200031 China

**Keywords:** IOL repositioning, Surgical prognosis, Tilt, Decentration

## Abstract

**Background:**

To compare the outcomes of sutured transscleral fixation and sutureless intrascleral fixation for the treatment of a dislocated intraocular lens (IOL).

**Methods:**

Thirty-five eyes of 35 patients who required IOL repositioning surgery due to IOL dislocation were included in this retrospective study. Sixteen eyes underwent two-point sutured transscleral fixation, eight eyes underwent one-point sutured transscleral fixation, and 11 eyes underwent sutureless intrascleral IOL fixation. The patients were followed for ≥ 12 months after repositioning surgery, and their postoperative outcomes were recorded and analyzed.

**Results:**

The major cause of IOL dislocation was ocular blunt trauma (19/35, 54.3%). The mean corrected distance visual acuity (CDVA) improved significantly after IOL repositioning (*P* = 0.022). The mean postoperative change in endothelial cell density (ECD) was − 4.5%. There were no significant differences in the changes in CDVA or ECD among the three groups with different repositioning techniques (both *P* > 0.1). The mean vertical tilt of the IOLs in all enrolled patients was significantly greater than the horizontal value (*P* = 0.001). The vertical tilt was greater in the two-point scleral fixation group than that in the sutureless intrascleral fixation group (*P* = 0.048). The mean decentration values in the one-point scleral fixation group in the horizontal and vertical directions were greater than those in the other two groups (all *P* < 0.01).

**Conclusion:**

All three IOL repositioning techniques resulted in favorable ocular prognosis.

## Background

Intraocular lens (IOL) dislocation is a serious postoperative complication of cataract surgery, and its frequency is escalating with the increasing numbers of patients undergoing cataract surgery [1–3]. Zonular weakness and an inadequate capsule are considered the main reasons for IOL dislocation, and are usually attributed to high myopia, vitreoretinal surgery, trauma, or retinitis pigmentosa [2, 4, 5].

In principle, in-the-bag IOL dislocation is treated with IOL exchange or IOL repositioning. IOL exchange may have a higher rate of complications because larger incisional wounds are made, whereas repositioning is performed in a closed system, and IOL exchange is more costly. In eyes with inadequate capsular support, the dislocated IOLs can be fixed to the iris or the sclera, for which a number of techniques have been described with advantages and disadvantages [6–9]. Surgeons may select the surgical technique according to their preference and experience.

In IOL repositioning, advantages of transscleral IOL suture technique include less corneal endothelial cell loss and fewer peripheral anterior synechiae, but suture-related complications are the main concern [10, 11]. For patients with a partial capsule, single-point transscleral IOL may be preferred because less maneuvering is required. The intrascleral IOL fixation technique was first reported by Gabor et al. [12] as a type of sutureless IOL fixation that avoids suture erosion or breakage. To date, however, few studies have compared the clinical outcomes of these surgical techniques, and the optimal management of late IOL dislocation is still contentious. In the present study, we compared the surgical outcomes and postoperative IOL positions among patients who underwent sutured transscleral fixation and sutureless intrascleral fixation.

## Methods

This retrospective study was approved by the Institutional Review Board of the Eye and Ear, Nose, and Throat (ENT) Hospital of Fudan University (no. 2021010-1). All procedures adhered to the tenets of the Declaration of Helsinki. Informed consent to participate in the study was obtained from all subjects. Informed consents for the use of medical data and publication of identifying information/images in an online open-access publication were also obtained from all subjects. All methods were carried out in accordance with relevant guidelines and regulations.

### Patients

Thirty-five eyes of 35 consecutive patients with late IOL dislocation who underwent IOL repositioning surgery at the Eye and ENT Hospital of Fudan University between January 2015 and January 2018 were recruited. The eyes were divided into three groups according to the repositioning method used: (1) sutureless intrascleral fixation (n = 11); (2) two-point sutured scleral fixation (n = 16); and (3) one-point sutured posterior chamber fixation (n = 8). The patients’ characteristics and preoperative data were reviewed and analyzed.

### Surgical technique

In 11 patients with a one-piece polymethyl-methacrylate or three-piece IOL with thin haptics, repositioning surgery was performed using the sutureless intrascleral fixation technique using the method described by Gabor and Agerwal [12, 13]. In 16 patients with a one-piece foldable IOL, two-point scleral fixation was performed using the *ab externo* suture loop closed-system fixation technique, as described by several other investigators [14, 15]. The other eight patients had partial capsular and zonular support, and the one-point scleral fixation technique was used, as previously described [16]. All procedures were performed by the same surgeon (C.H.J.).

### Postoperative examinations

At the final follow-up, all patients underwent thorough ophthalmological examinations, including uncorrected visual acuity, corrected distance visual acuity (CDVA), intraocular pressure (IOP), noncontact specular microscopy, and slit-lamp biomicroscopy examinations. Snellen visual acuity measurements were converted to logarithm of the minimum angle of resolution (logMAR) equivalents for data analysis. Anterior segment tomography (Pentacam HR, Oculus, Wetzlar, Germany) was performed before and after mydriasis. IOL decentration and tilt were measured as described in our previous study [17].

### Statistical analysis

All analyses were performed with SPSS version 19.0 (SPSS, Chicago, IL, USA). Categorical values were expressed as proportions and continuous variables were expressed as means ± standard deviations (SD). Shapiro-Wilk test was applied to check the normality of the data before t-test, and P value > 0.05, which indicated that the assumption of a normal distribution had not been violated. Student’s t test was used to examine variables between two groups. One-way analysis of variance (ANOVA) with a post-hoc Tukey HSD test was used to examine variables among three groups. Paired *t* tests were used to compare the preoperative and postoperative variables. *P* values of ≤ 0.05 were considered statistically significant.

## Results

Thirty-five patients were included in the study, and 26 (74.3%) were male. Their mean age at the time of repositioning surgery was 53.51 ± 15.69 years and the mean time from repositioning surgery to the last follow-up visit was 22.55 ± 9.03 months (range 12–42 months). The mean axial length was 24.98 ± 2.54 mm. The 35 patients were divided into three groups according to different surgical techniques used, and the baseline characteristics of the three groups are shown in Table 1. There were no significant differences among the three groups in terms of age, and the time interval between cataract surgery and IOL dislocation (all P > 0.1, one-way ANOVA with post-hoc Tukey test for pairwise comparisons). The axial length was greater in the two-point scleral fixation group than in the sutureless intrascleral-fixed group (P = 0.011), but it was not significantly different from that in the one-point scleral fixation group (P = 0.093, one-way ANOVA with post-hoc Tukey test for pairwise comparisons).

**Table 1:** Patient characteristics

Parameter		Sutureless intrascleral fixation	Two-point sutured transscleral fixation	One-point sutured transscleral fixation
Sex (M/F)		10/1	8/8	8/0
Operated eye (R/L)		5/6	8/8	4/4
Age (years)		50.55 ± 19.52	58.38 ± 14.18	47.88 ± 10.75
Axial length (mm)		23.71 ± 1.52	26.21 ± 2.32	24.44 ± 1.64
Interval between cataract surgery and IOL dislocation (years)		6.11 ± 4.92	5.47 ± 4.33	5.91 ± 4.47
Follow-up duration (months)		22.27 ± 7.58	22.71 ± 10.01	22.63 ± 10.23
Type of IOL	Foldable one-piece IOL	0	16	5
	Three-piece IOL or single-piece PMMA IOL	11	0	3

M, male; F, female; R, right; L, left; IOL, intraocular lens; PMMA, polymethyl-methacrylate

Of the 35 eyes assessed for eligibility, 19 eyes (54.3%) had a history of ocular blunt trauma. Other causes of IOL dislocation were previous vitreoretinal surgery (n = 4, 11.4%), high myopia (n = 6, 17.1%), and Nd:YAG (Neodymium-doped Yttrium-Aluminum Garnet) laser capsulotomy (n = 1, 2.9%). Five eyes (14.3%) were diagnosed with age-related cataract, with no known predisposing condition, and underwent regular phacoemulsification with IOL implantation.

The mean CDVA of the patients improved from 0.78logMAR to 0.65logMAR at the last visit (*P* = 0.022, paired *t* test). The mean endothelial cell density (ECD) decreased by about 4.5% (from 2298.93 ± 506.53/mm^2^ to 2194.31 ± 544.84/mm^2^, *P* < 0.001, paired *t* test). The mean IOP remained unchanged (pre-surgery 16.6 ± 5.3 mmHg; last visit 16.39 ± 3.71 mmHg, *P* = 0.806, paired *t* test). There were no significant differences among the three groups in terms of the changes in CDVA, ECD, or IOP (all *P* > 0.1, Table 2.).

**Table 2:** Clinical outcomes in the three groups

Variable	Sutureless intrascleral fixation	Two-point sutured transscleral fixation	One-point sutured transscleral fixation
Postoperative IOP (mmHg)	16.15 ± 2.31	15.77 ± 2.89	17.94 ± 6.14
Change in CDVA (logMAR) (last visit − preoperative visit)	−0.19 ± 0.27	−0.15 ± 0.26	−0.29 ± 0.43
Change in ECD (cells/mm^2^) (last visit − preoperative visit)	−104.57 ± 100.30	−96.57 ± 72.82	−121.00 ± 119.78

IOP, intraocular pressure; CDVA, corrected distance visual acuity; logMAR, logarithm of the minimum angle of resolution; ECD, endothelial cell density; IOL, intraocular lens

Six patients suffered transient elevated IOP (> 25 mmHg) postoperatively and eye drops were administered to lower their IOP. Of these patients, one with a history of ocular trauma underwent one-point sutured transscleral fixation. Two patients with a history of vitreoretinal surgery and one with ocular trauma underwent two-point sutured transscleral fixation. One patient with a history of vitreoretinal surgery and one with a history of ocular trauma underwent sutureless intrascleral fixation.

**Fig. 1:**
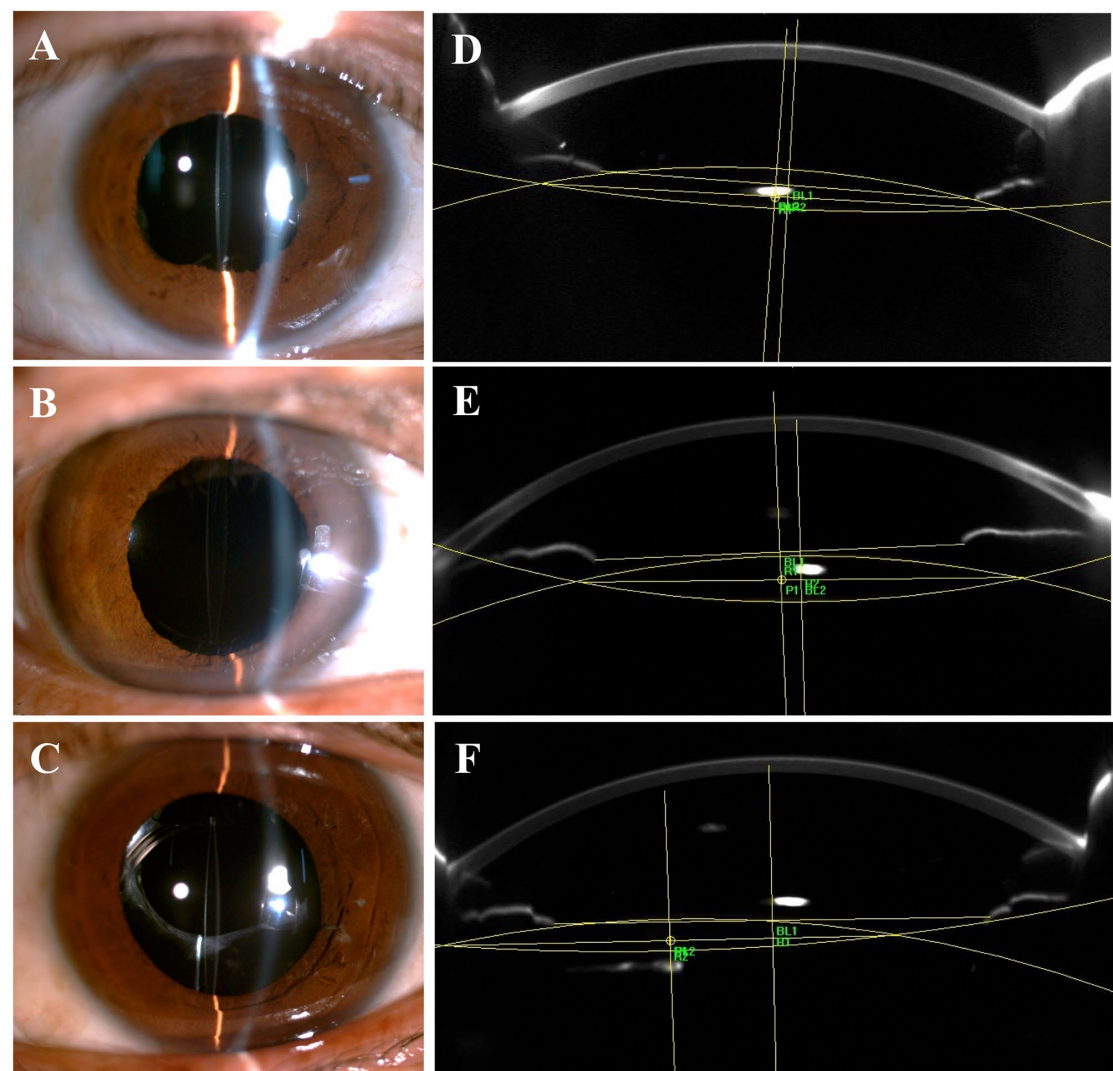
Anterior segment photographs and illustrations of the vertical tilt and decentration measurements with Scheimpflug images of patients from the three groups. A&D: A two-point sutured IOL with a vertical tilt of 2.74° and decentration length of 0.31 mm. B&E: Sutureless intrascleral IOL fixation with a vertical tilt angle of 1.95° and decentration length of 0.28 mm. C&F: One-point (5 o’clock) sutured IOL fixation. The IOL is slightly eccentric with a vertical tilt angle of 1.19° and a decentration length of 1.11 mm

Among all 35 patients, the mean horizontal tilt angle was 1.91°± 1.60° with a decentration length of 0.35 ± 0.45 mm. The mean vertical tilt was 3.45°± 2.33° with a decentration length was 0.45 ± 0.46 mm. The vertical tilt was significantly greater than the horizontal tilt (*P* = 0.001, paired *t* test). As shown in Table 3, the vertical tilt was greater in the two-point scleral fixation group than in the sutureless intrascleral-fixed group (*P* = 0.048), but it was not significantly different from that in the one-point scleral fixation group (P = 0.523, one-way ANOVA with post-hoc Tukey test for pairwise comparisons). The horizontal tilt was similar in all three groups (all P > 0.1, one-way ANOVA with post-hoc Tukey test for pairwise comparisons). There were no significant differences in the horizontal and vertical decentration lengths between the two-point scleral fixation and sutureless fixation groups (both P > 0.4, Student’s t test). The mean decentration lengths in both the horizontal and vertical directions in the one-point scleral fixation group were greater than those in the other two groups (all P < 0.01, one-way ANOVA with post-hoc Tukey test for pairwise comparisons; Table 3.). Typical anterior segment photographs and the corresponding vertical Scheimpflug images of patients from the three groups are shown in Fig. 1.

**Table 3:** IOL tilt and decentration lengths in the three groups

Variable	Sutureless intrascleral fixation	Two-point sutured transscleral fixation	One-point sutured transscleral fixation
Horizontal tilt (°)	1.79 ± 1.43	1.60 ± 1.27	2.67 ± 2.27
Vertical tilt (°)	2.35 ± 2.16	4.17 ± 2.33*	3.54 ± 2.25
Horizontal decentration (mm)	0.28 ± 0.34	0.24 ± 0.29	0.81 ± 0.63*
Vertical decentration (mm)	0.30 ± 0.26	0.38 ± 0.30	0.87 ± 0.72*

*P < 0.05, one-way ANOVA with post-hoc Tukey method for pairwise comparisons

## Discussion

The incidence of IOL dislocation has increased in the past decade [18, 19]. The choice of surgical method generally depends on the surgeon’s preference. Previous studies have shown that sutureless intrascleral IOL fixation has become a popular procedure because it has several advantages over conventional transscleral suturing of IOLs [20–23]. However, no studies have compared the efficacy of these approaches or evaluated the tilt and decentration of these IOLs. In the present study, no severe complications were observed other than a transient elevation of IOP. Satisfactory visual outcomes and IOP positions were achieved in all three groups, although two-point scleral fixation resulted in greater vertical tilt and one-point scleral fixation resulted in greater decentration.

We compared the baseline characteristics among three groups and found a significant difference in axial length between two-point scleral fixation and sutureless intrascleral-fixed group. Two-point scleral fixation was chosen in myopic subjects due to the fact that patients with long axial length, the sclera was usually thinner and sutureless intrascleral fixation might not be a proper choice.

In this study, the follow-up CDVA increased significantly in all enrolled patients although the mean postoperative CDVA of 0.65logMAR was less favorable than the postoperative CDVA of 0.3–0.5logMAR reported in previous studies [24–27]. The types of predisposing conditions and the ocular comorbidities may preclude direct comparisons among these studies. A history of blunt trauma, high myopia, and vitreoretinal surgery were common causes in the present study, whereas pseudoexfoliation appeared to be the predominant factor in the earlier studies [2, 5, 28]. In the present study, patients with a history of blunt trauma or vitreoretinal surgery seemed to have worse postoperative visual outcomes.

A long surgical period and complicated surgical manipulations result in the loss of endothelial cells during IOL repositioning procedures. Kristianslund et al. [28] reported that postoperative ECD loss was significantly greater after IOL exchange (10%) than after IOL repositioning (3%), whereas Oh et al. [4] reported that the change in ECD was not significantly different between patients who underwent IOL exchange (5.93%) and patients who underwent IOL repositioning (5.09%). In the present study, the mean endothelial cell density decreased by 4.5%, and no patient developed corneal endothelial decompensation. We used dispersive viscosurgical device during the IOL repositioning surgery to protect the corneal endothelium. And prednisolone acetate and recombinant human epidermal growth factor derivative eye drops were prescribed postoperatively to reduce corneal edema.

A common complication of all IOL repositioning operations is that the posterior IOL may be tilted and decentered after surgery, leading to visual disturbances. In this study, the mean postoperative vertical tilt angle was 3.45°, which was significantly greater than the horizontal tilt angle of 1.91°. Yamane et al. [29] previously reported that the mean IOL tilt was 3.4°± 2.5° following posterior chamber sutureless implantation. In a study by Hayashi et al. [22] who measured the tilt and decentration of scleral-sutured IOLs, the mean tilt angle was 6.35° and the decentration length was 0.62 mm. Here, we compared the results of three different surgical methods for the first time. In the two-point scleral-sutured fixation group, the tilt in the vertical direction was greater than that in the horizontal direction (*P* = 0.001), whereas decentration was similar in both directions (mean vertical decentration was 0.24 mm and mean horizontal decentration was 0.38 mm). In the two-point sutured transscleral fixation group, the IOL haptics were fixed at 3 o’clock and 9 o’clock, but the IOLs were not fixed in the vertical direction. This would have been responsible for the greater tilt in the vertical direction. IOL tilt may lead to blurred vision, including astigmatism, myopic shift, and uncorrectable coma aberration [30]. Therefore, a technique that involves three or more points of fixation may reduce the risk of IOL tilt [31].

The intrascleral fixation technique was introduced by Gabor in 2007 [12]. However, as early in 1997, Teichmann et al. [32] had suggested that the longer part of the haptic should stabilize the axial position of a posterior chamber IOL and reduce the risk of IOL tilt. In the present study, although both groups had greater tilt in the vertical direction than the horizontal direction, the tilt was smaller in the sutureless fixation than in the two-point sutured fixation group. However, we did not perform this procedure in patients with high myopia, in deference to the thin sclera in these patients. Therefore, the axial length was greater in patients who underwent two-point transscleral IOL fixation (26.21 ± 2.32 mm) than in those who underwent sutureless fixation (*P* < 0.01).

In single-point transscleral IOL fixation, one haptic was placed on the residual capsule, so the position of the IOL was determined by more than two points, and the tilt in both the horizontal and vertical directions was small. However, this group had greater decentration in both the horizontal and vertical directions, which was in accordance with our clinical impression that the IOL was decentered in the direction of the suture.

The fixed part of IOL and lens type would have influences on the position of the IOL. In our experience, the fixed part tends to be fixed at the distal end of the IOL loop. In one-point scleral fixation group, the IOL tends to be decentered to the direction of fixation. In reference to the lens type, three-piece IOLs are prone to postoperative IOL pupillary capture, which affects the IOL position, while foldable one-piece IOL with closed C-loop are not prone to this condition. Our study was limited by its small sample size, and further study with a large sample size is needed.

## Conclusion

The surgical efficacy of all three IOL repositioning techniques seemed similar in terms of the visual prognosis, ECD damage, and IOP. To reduce the tilt of the IOL, a technique involving fixation at three or more points should be considered. But our cases and follow-up period were still limited, future study was still required to improve our knowledge in this field.

## Data Availability

The data, which include patient privacy information, are not deposited publicly due to ethical reason, and are available from the corresponding author upon request.

## Abbreviations

IOL: Intraocular lens
CDVA: Corrected distance visual acuity
ECD: Endothelial cell density
IOP: Intraocular pressure
logMAR: Logarithm of the minimum angle of resolution
SD: Standard deviation
PMMA: Polymethyl-methacrylate
NdYAG: Neodymium-doped Yttrium-Aluminum Garnet
